# Supplementary material for: A Mycobacterium tuberculosis Effector Targets Mitochondrion, Controls Energy Metabolism, and Limits Cytochrome c Exit
Source: Microbiol Spectr. 2023 Apr 10;11(3):e01066-23. doi: 10.1128/spectrum.01066-23 (PMC10269737; doi:10.1128/spectrum.01066-23)
Supplement: Supplemental file 1 — Supplemental legends. Download spectrum.01066-23-s0001.pdf, PDF file, 0.10 MB [file spectrum.01066-23-s0001.pdf]

SUPPLEMENTARY DATA LEGENDS

Fig. S1: Rv1813 is detected in *M. tuberculosis* culture filtrate but not in cellular fractions. Lysate obtained from *M. tuberculosis* H37Rv strain grown in Sauton's medium to logarithmic phase (OD_{580}~0.7) were fractionated and probed with anti-Rv1813 polyclonal antibodies. Culture filtrates were obtained from the same culture. Anti-FtsZ (FtsZ is a cytoplasmic protein), anti-GlnA (GlnA is a membrane protein) and anti-RpfB (RpfB is a membrane and cell-wall anchored protein) antibodies were used to confirm the purity of mycobacterial fractions.

Fig. S2: ^{1}H-^{15}N HSQC spectrum of Rv1813c

This spectrum was obtained at 800 MHz, 20°C and pH 6,8 with 0.3 mM ^{15}N-uniformly labeled sample. Cross peak assignments are indicated using the one-letter amino acid code and number following the full-length protein sequence numbering.

Fig. S3: Confocal microscopy analysis of Rv1813c family members localization in *Dictyostelium.*

Dictyostelium cells expressing the indicated constructs were fixed, processed for immunofluorescence, and analyzed by confocal microscopy (Airyscan).

(A) Cellular localization of Nt-GFP-tagged Rv1813c. **(B)** Mitochondrial localization of untagged Rv1813c expressed in *Dictyostelium*. Rv1813c was labeled with a rabbit pAb anti-Rv1813c antibody. Rv1813c colocalizes with Mitoporin, a mitochondrial specific protein. **(C)** Mitoporin localization in untransfected parental Ax2 cells. Cells were labeled with a mouse mAb to mitoporin revealing characteristic ring shaped structures. A maximum projection of Z confocal sections is shown on the right panel. **(D)** Localization of different Rv1813c family members of *M. tuberculosis* and *M. marinum* expressed in *Dictyostelium and* revealed by anti-myc labelling. White arrows indicate so mitochondria with affected shapes. Bar, 5 µm.

Fig. S4: Rv1813c family localization in HeLa cells

HeLa cells expressing the indicated constructs were fixed, processed for immunofluorescence, and analyzed by confocal microscopy (Airyscan). Cells were colabeled either with rabbit polyclonal anti-Rv1813c, mouse mAb anti-Cytochrome c, and mitotracker deep red (upper panel) or anti-myc, rabbit anti-grp75 (mitochondria marker) and mitotracker deep red (lower panels). Bar, 10 µm.

Supplementary table 1: NMR and refinement statistics for RV1813 protein structures
