# Supplementary figures and images for: A Mycobacterium tuberculosis Effector Targets Mitochondrion, Controls Energy Metabolism, and Limits Cytochrome c Exit
Source: Microbiol Spectr. 2023 Apr 10;11(3):e01066-23. doi: 10.1128/spectrum.01066-23 (PMC10269737; doi:10.1128/spectrum.01066-23)

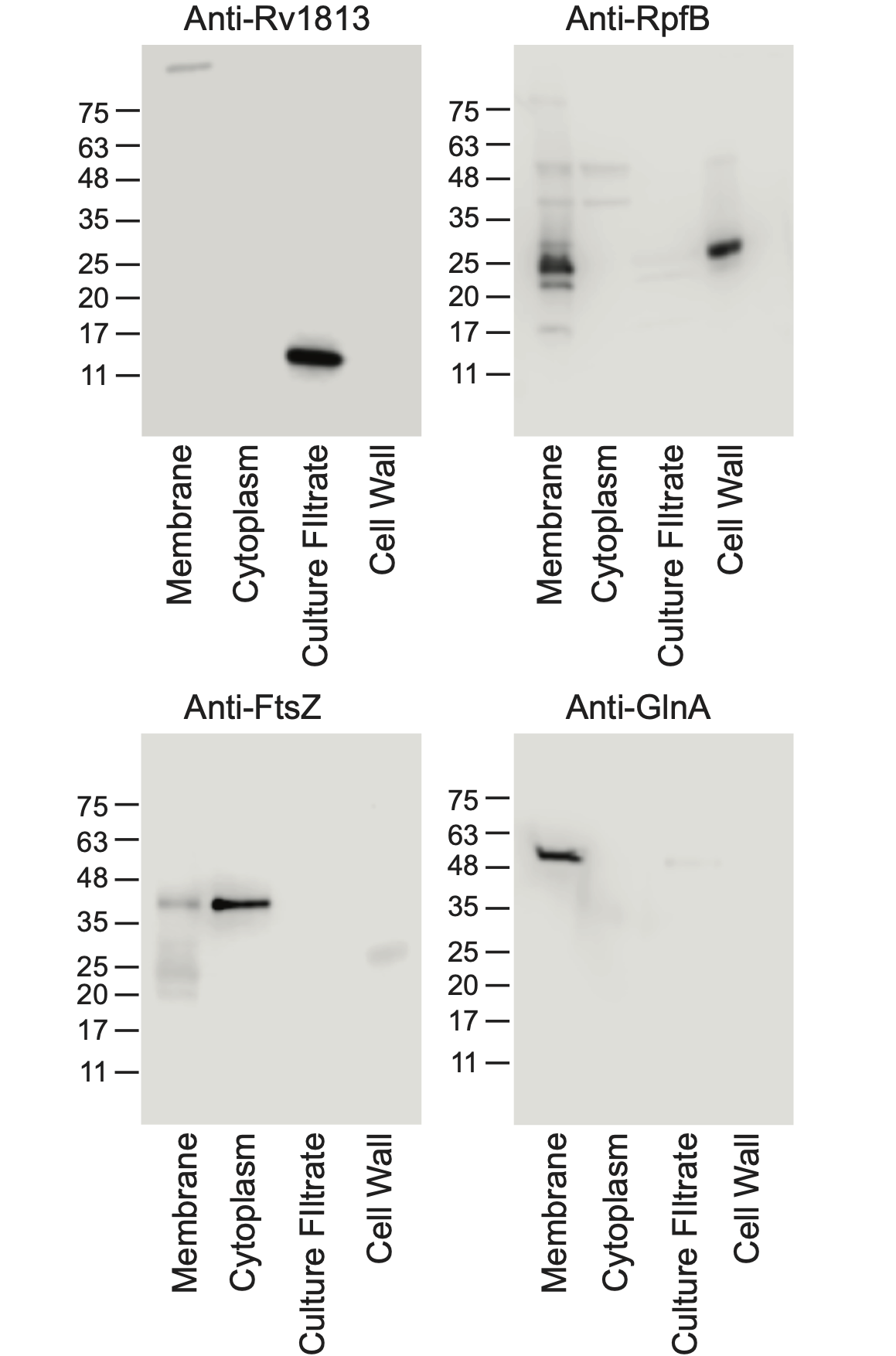

Supplement: Supplemental file 2 — Fig. S1. Download spectrum.01066-23-s0002.tif, TIF file, 7.8 MB [file spectrum.01066-23-s0002.tif]

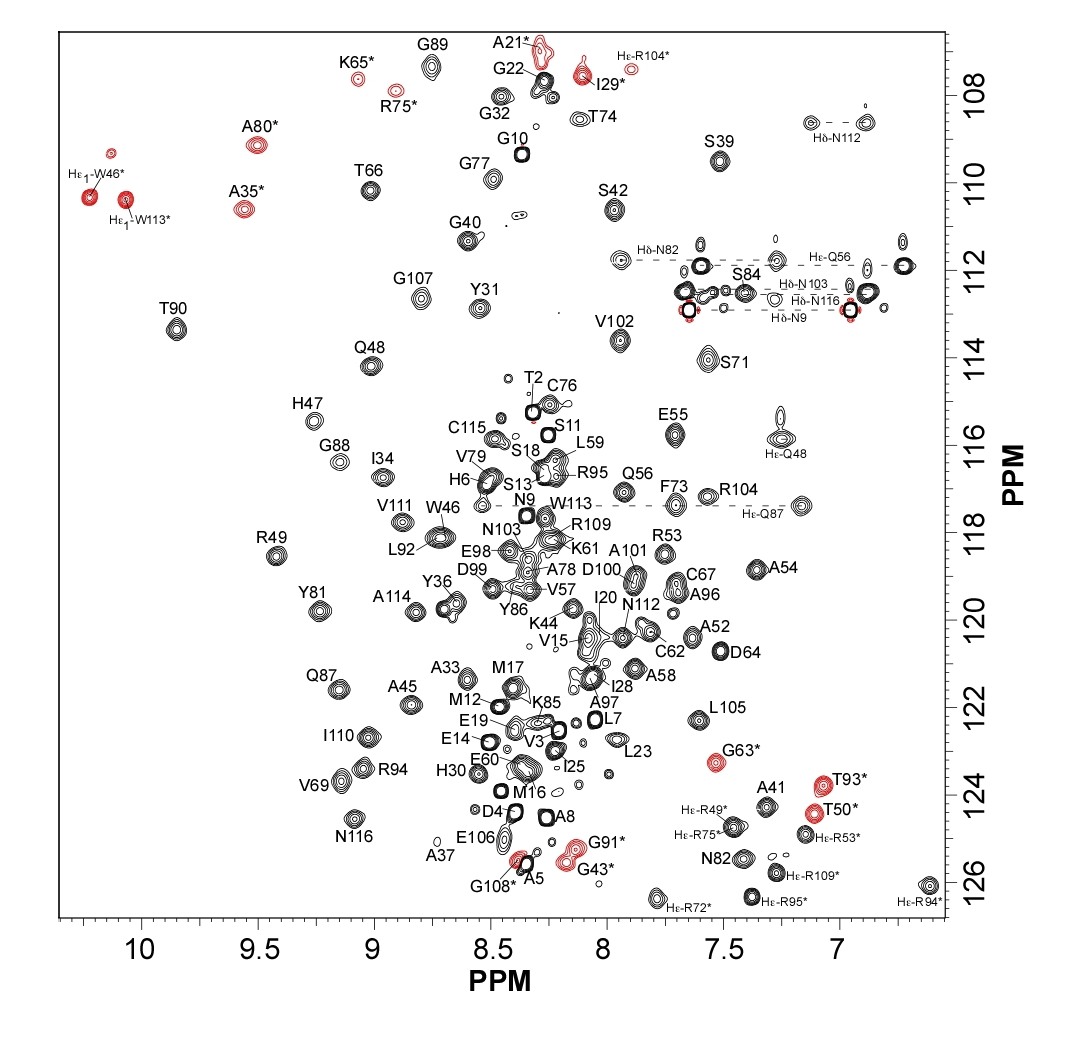

Supplement: Supplemental file 3 — Fig. S2. Download spectrum.01066-23-s0003.tif, TIF file, 4.4 MB [file spectrum.01066-23-s0003.tif]

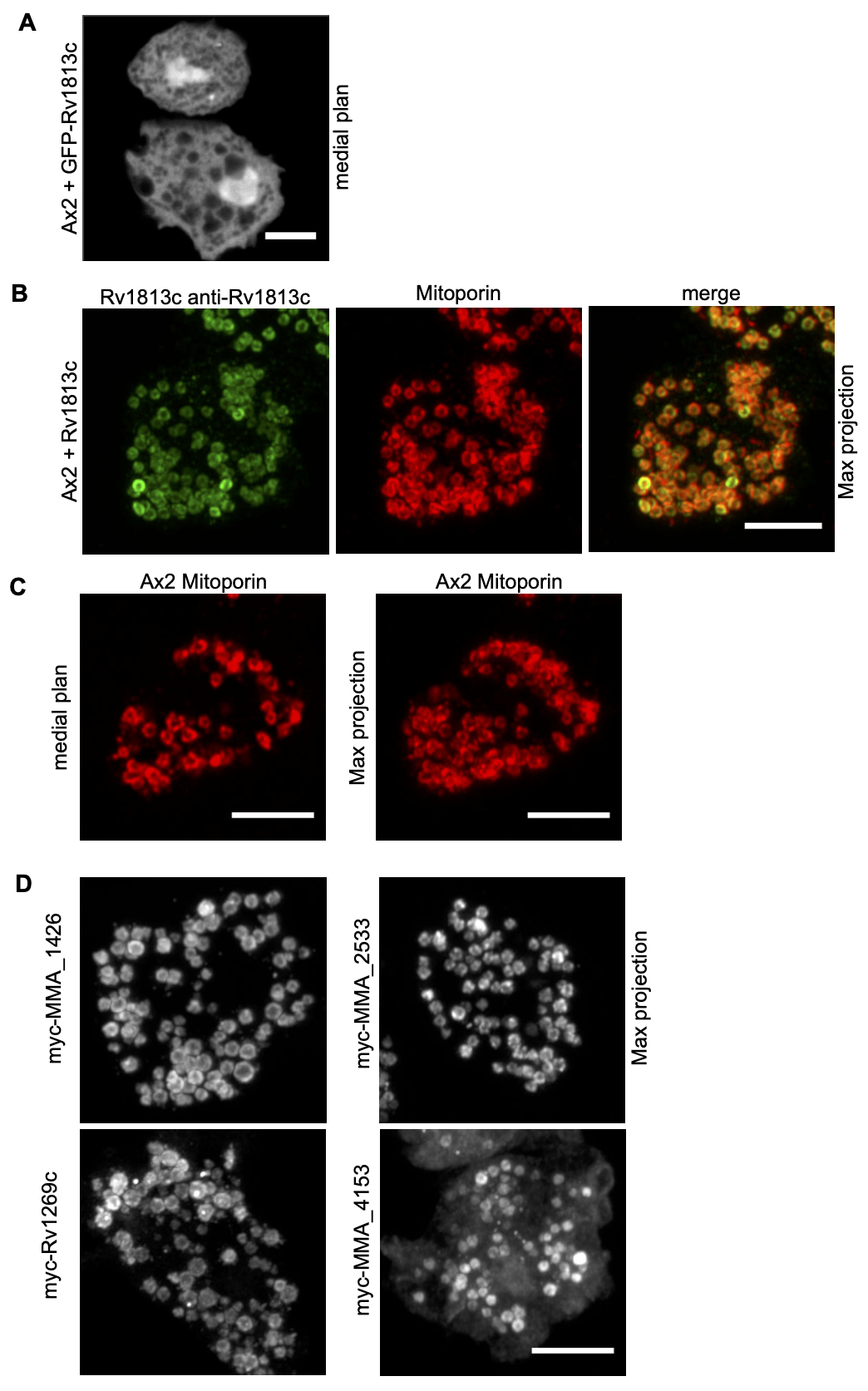

Supplement: Supplemental file 4 — Fig. S3. Download spectrum.01066-23-s0004.tif, TIF file, 6.7 MB [file spectrum.01066-23-s0004.tif]

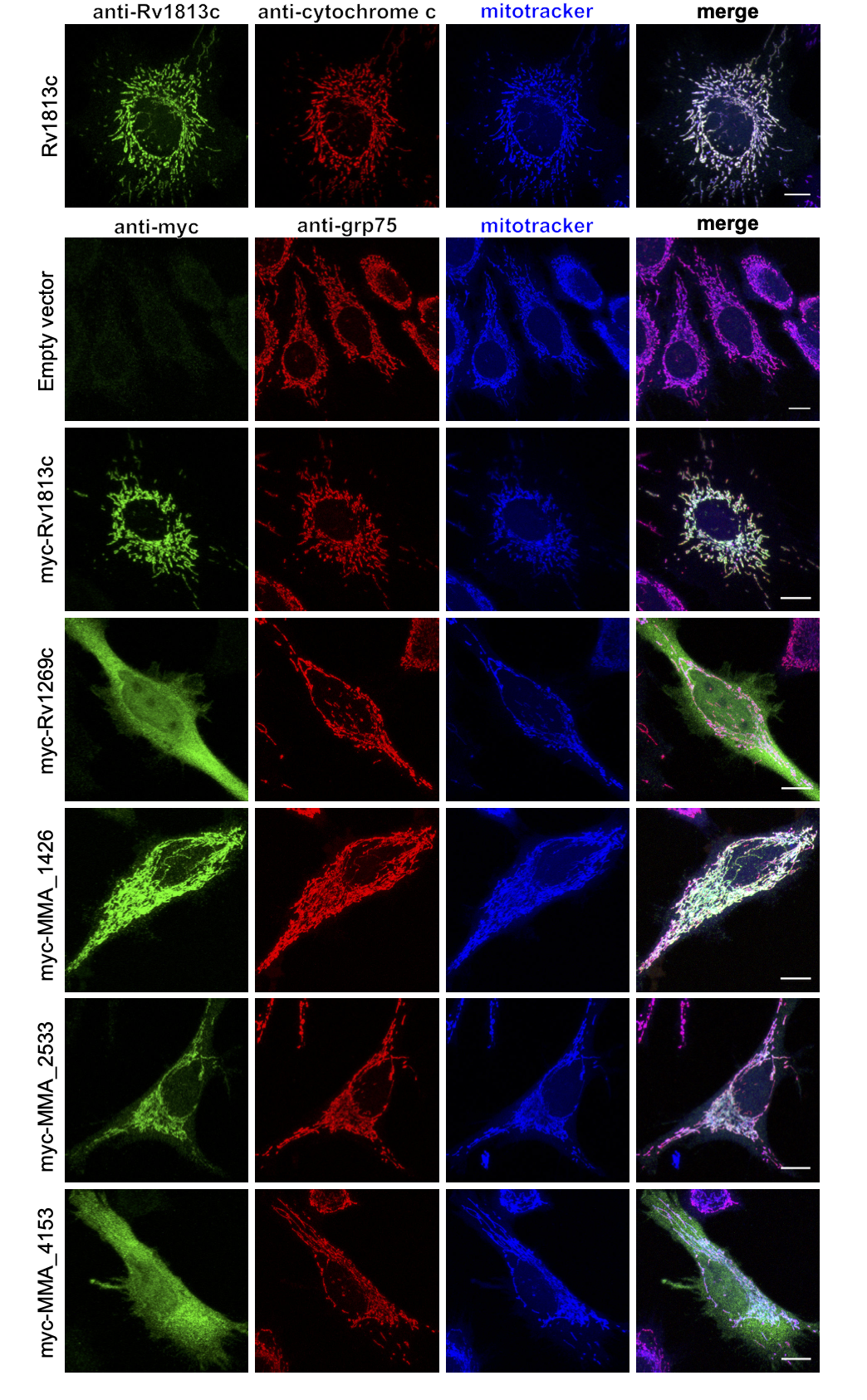

Supplement: Supplemental file 5 — Fig. S4. Download spectrum.01066-23-s0005.tif, TIF file, 6.7 MB [file spectrum.01066-23-s0005.tif]
