# Supplementary material for: A Mycobacterium tuberculosis Effector Targets Mitochondrion, Controls Energy Metabolism, and Limits Cytochrome c Exit
Source: Microbiol Spectr. 2023 Apr 10;11(3):e01066-23. doi: 10.1128/spectrum.01066-23 (PMC10269737; doi:10.1128/spectrum.01066-23)
Supplement: Supplemental file 6 — Table S1. Download spectrum.01066-23-s0006.docx, DOCX file, 0.02 MB [file spectrum.01066-23-s0006.docx]

**SUPPLEMENTARY TABLE 1**

**NMR and refinement statistics for RV1813 protein structures**

**NMR distance and dihedral constraints**

Distance constraints

Total NOE 1516

Intra-residue 406

Inter-residue

Sequential (|i – j| = 1) 465

Medium-range (|i – j| < 4) 253

Long-range (|i – j| > 5) 392

Hydrogen bonds 84

Total dihedral angle restraints

φ 82

ψ 82

**Structure statistics**

Violations (mean and s.d.)

Max. distance constraint violation (Å) 0.18 ± 0.03

Max. dihedral angle violation (º) 2.04 ± 0.48

Deviations from idealized geometry

Bond lengths (Å) 0.0118 ± 0.0002

Bond angles (º) 1.2010 ± 0.0214

Impropers (º) 1.3446 ± 0.0861

**Ramachandran plot (%)**

Most favoured region 84.7

Additionally allowed region 14.2

Generously allowed region 0.8

Disallowed region 0.3

**Average pairwise r.m.s. deviation** (Å)**

Backbone 0.66 ± 0.18

Heavy 1.26 ± 0.19

** “Pairwise r.m.s. deviation calculated among 20 refined structures for residues 31-116.”
